# Adaptation to Leaf Traits of Individual Trees in a Forest Appears Rare in Caterpillars

**DOI:** 10.1002/ece3.71038

**Published:** 2025-03-13

**Authors:** Freerk Molleman, Anna Sokół‐Łętowska, Soumen Mallick, Andreas Prinzing, Urszula Walczak

**Affiliations:** ^1^ Department of Systematic Zoology, Institute of Environmental Biology, Faculty of Biology Adam Mickiewicz University in Poznań Poznań Poland; ^2^ Department of Fruit, Vegetable and Plant Nutraceutical Technology, Faculty of the Biotechnology and Food Science Wrocław University of Environmental and Life Sciences Wrocław Poland; ^3^ Field Station Fabrikschleichach, Department of Animal Ecology and Tropical Biology, Biocenter University of Würzburg Würzburg Germany; ^4^ Research Unit Ecosystemes, Biodiversité, Evolution Université de Rennes 1, Centre National de la Recherche Scientifique Rennes France

**Keywords:** adaptive deme, herbivorous insect, local adaptation, phylogenetic isolation, plant defense, tree diversity

## Abstract

High herbivore abundances on trees surrounded by distantly related neighbors (phylogenetic isolation) might in part be due to local adaptation of herbivores to host trees, but this has not been tested. We studied if free‐feeding and semi‐concealed (shelter‐building) Lepidoptera can be adapted to leaf traits of individual trees, and if this is affected by phylogenetic isolation. We performed a reciprocal transplant experiment on free‐feeding and semi‐concealed lepidopteran caterpillars collected from oak trees ( *Quercus petraea* ) in a mixed forest in Poland. Within a set of trees with early and a set with late budburst, we selected oak trees that varied from being surrounded by other oak trees (low phylogenetic isolation) to oaks surrounded by pine trees (high phylogenetic isolation), and collected canopy branches to obtain caterpillars. We then fed half of the caterpillars leaves from the tree they were collected from (home tree) and others on the leaves of another tree in the set (away trees) in the laboratory. We measured caterpillar mass over a five‐day interval to calculate growth rate and determined aspects of leaf chemistry of each tree. Five species of Lepidoptera (*Acrobasis repandana, Eudemis profundana, Operopthera brumata, Phycita roborella, Zeiraphera isertana*) yielded sufficient sample sizes for statistical analyses. Overall, we found faster growth on home trees, which could be attributed to one species, *E. profundana*. There was no effect of phylogenetic isolation. Our results indicate that local adaptation to leaf traits of individual trees is rare in these lepidopterans, and we found no evidence that local adaptation would be more pronounced on trees that are more phylogenetically isolated from their neighbors. Therefore, the effects phylogenetic isolation on herbivory are not likely to be mediated by local adaptation to individual trees.

## Graphical abstract

We performed a reciprocal transplant experiment in which we reared half of the caterpillars collected from large oak trees in a forest on leaves from the home tree, and the rest on leaves of the other oak trees. We found that in one species, the growth rate was higher on leaves of the home tree, indicating adaptation to the individual trees.

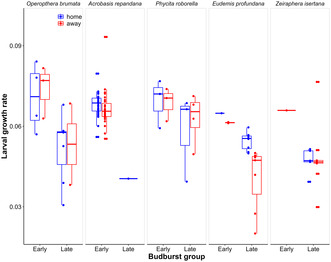

## 1 Introduction

Individual trees within a population can display consistent differences from each other in phenology, leaf nutritional quality, and defenses (e.g., Eisenring et al. 2021; Tikkanen and Julkunen‐Tiitto 2003), which may cause local adaptations in herbivorous insect populations. Such local adaptation (also known as adaptive‐deme formation) can be defined as insects performing better on home trees than on other conspecific trees of the same population, without knowledge of what particular traits of trees the insects are adapted to. However, it remains unclear what tree and insect characteristics promote this. Such local adaptations have been shown in scale insects (Edmunds and Alstad 1978), leaf miners (Mopper et al. 2000; Tack and Roslin 2010), a leaf roller and a galler (Tack and Roslin 2010), and in moths with wingless females (Van Dongen et al. 1997). In contrast, other studies have not found such local adaptation (scale insects: Cobb and Whitham 1993; sawflies: Ruhnke et al. 2006). Local adaptation over multiple generations requires that insect populations are somewhat isolated on individual trees and is thus more likely in more sedentary insects (Boecklen and Mopper 1998; Zandt and Mopper 1998). Nevertheless, even winged insects can form local populations, such as when leaf rollers mate and reproduce near the tree they developed on (Schneider 1984). Such local populations appear to be unusual; however, even for small leaf miners (Connor et al. 1983). To our knowledge, direct tests for such local adaptation in larger (winged) Lepidoptera are lacking. Correlations between the abundance of caterpillars and their parasitism rates at the level of individual trees do suggest that some flying Lepidoptera with semi‐concealed (shelter‐building) or free‐feeding caterpillars might form populations at such small scales (Molleman et al. 2022). This could promote local adaptation in these insects, but this has not been previously demonstrated.

Isolation of trees may promote local adaptation in insects as it reduces the influx of non‐adapted individuals. Indeed, Tack and Roslin (2010) found that herbivorous insects on free‐standing *Quercus robur* trees were more likely to show such local adaptation than those surrounded by conspecific neighbors. Individual trees can also be isolated in mixed forest stands. In particular, phytophagous arthropods feeding on leaves of trees in a mixed forest can usually use only a limited set of tree species (Novotny et al. 2004; Southwood and Kennedy 1983). For such specialized arthropods, individual trees in a mixed forest form habitat islands. Since host‐plant relations of arthropods tend to have a phylogenetic signal (Brändle and Brandl 2006; Ehrlich and Raven 1967), this islandness of individual trees (defined as being difficult to reach for specialized phytophagous arthropods) can be represented by phylogenetic isolation (average crown phylogenetic distance to neighboring trees; Vialatte et al. 2010). In practical terms, for specialized phytophagous arthropods in a forest, the degree of phylogenetic isolation of an individual tree can range from being surrounded by conspecifics (zero phylogenetic isolation) to being surrounded by distantly related tree species (high phylogenetic isolation). Phylogenetic isolation has been shown to decrease herbivory on small trees (Yguel et al. 2011), but to increase herbivore abundance on large trees (Molleman et al. 2022). Higher herbivore abundance may in part be due to local adaptation in insect populations on more isolated trees. Such local adaptation is also indicated by the finding that on more phylogenetically isolated trees, individuals of root‐feeding click beetle species tend to be larger as they face less (indirect) competition as larvae, and individuals of species with predacious larvae tend to be smaller as they may experience lower prey density (Molleman et al. 2016). However, direct tests for adaptation of herbivorous insects to individual trees that vary in phylogenetic isolation have so far not been reported.

Adaption to an individual tree can potentially be to various traits such as budburst phenology, leaf nutritional properties, defenses, and even microclimate. To our knowledge, budburst phenology is the only trait that was specifically studied in tests for adaptation to individual trees (Serra et al. 2019; Van Dongen et al. 1997). For insect species that rely on synchronizing their phenology with that of their host, such phenology matching undoubtedly has very strong fitness consequences (Feeny 1970; Meineke et al. 2021), so that it may be difficult to sample non‐matching individuals from trees as they would die or move away. Tests for adaptations to phenology thus typically focus on synchrony between egg hatching and budburst. In addition to extensive variation in budburst between individual trees (Heinecke et al. 2024), there is also extensive variation in leaf traits such as nutritional quality and defensive chemistry (Bertić et al. 2021; Eisenring et al. 2021; Valdés‐Correcher et al. 2020), but local adaptation of insects to such leaf traits has not been addressed separately from budburst phenology. However, adaptation to budburst seems less likely in species that develop later in the season such as many species of leaf miner, and such cases are thus likely cases of adaptation to leaf traits (Mopper et al. 2000). As leaf nutritional quality (such as captured by C:N ratio) and defenses (such as certain polyphenols) affect caterpillar growth rate (Feeny 1968), herbivore populations might become adapted to such leaf traits (Azevedo‐Schmidt & Currano, 2024).

**FIGURE 1:**
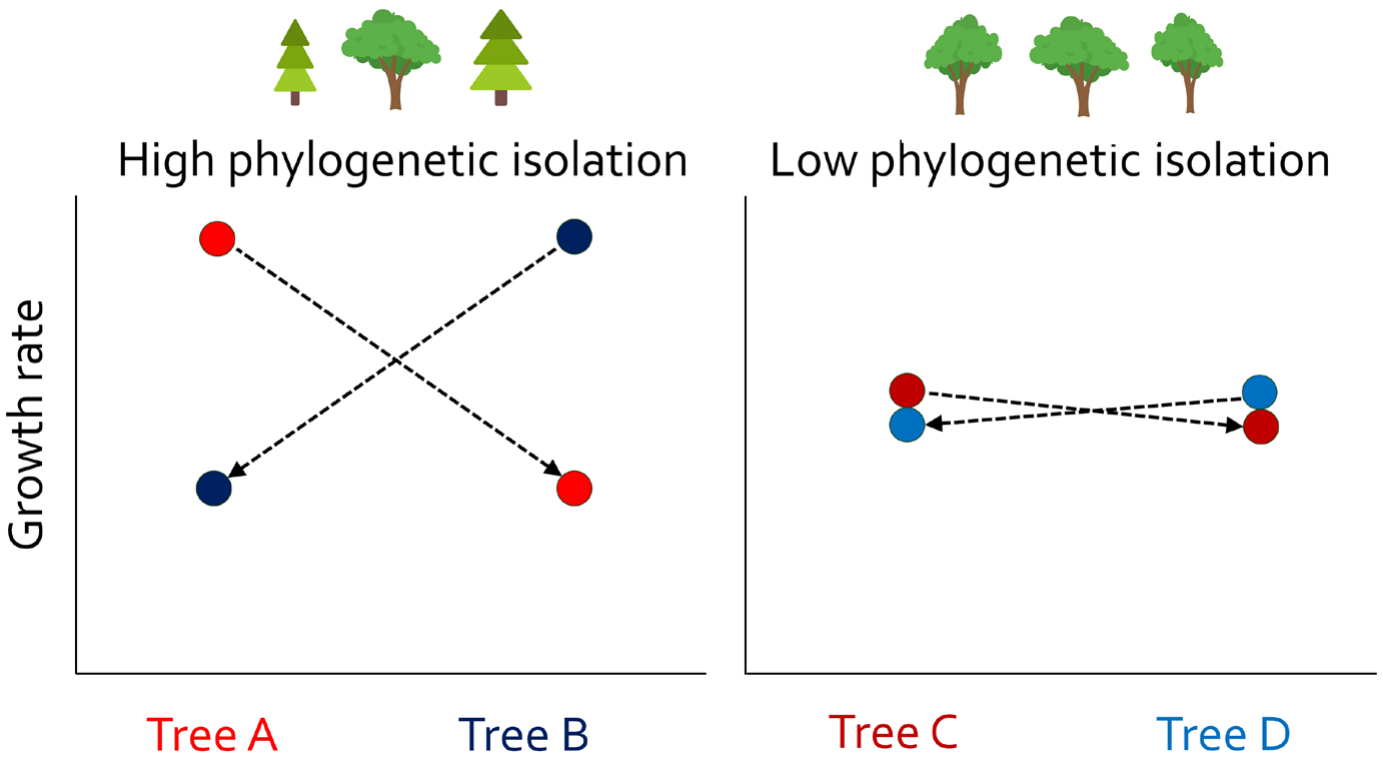
Our hypotheses, where circles denote the growth rate of caterpillars feeding on the home tree (matching colors between tree and circle) and away trees (non‐matching colors). Under high phylogenetic isolation, we expect that caterpillars perform better on the home tree than on away trees, and when phylogenetic isolation is low, we expect this effect to be smaller.

We performed a reciprocal transplant experiment on free‐feeding (*Operopthera brumata*) and semi‐concealed (*Acrobasis repandana, Eudemis profundana, Phycita roborella, Zeiraphera isertana*) Lepidopteran caterpillars from large oak trees ( *Q. petraea* ) that varied in their degree of phylogenetic isolation. Caterpillars on the same trees previously showed correlations between parasitism and caterpillar abundance at the level of individual trees, suggesting local parasitoid‐host dynamics and thus indicating local populations (Molleman et al. 2022). We tested the hypotheses that (a) these Lepidoptera show local adaptation to tree traits other than budburst phenology, and (b) local adaptation is more pronounced on more phylogenetically isolated trees (Figure 1). We also accounted for carbon‐to‐nitrogen ratio (C:N) and concentrations of polyphenols as possible leaf traits that are likely to affect growth rate (Feeny 1968; Forkner et al. 2004; Lindroth et al. 2024). We collected caterpillars from two sets of trees with similar budburst phenology within a set, and reared half on leaves from the tree from which they were collected (“home” tree) and the others on leaves of other trees in the set (“away” tree). We measured fitness as caterpillar growth rate, expecting a higher growth rate on home trees than on away trees if caterpillars were locally adapted. Since caterpillar growth rate can be increased or decreased by pathogens and parasitoids (Cuny and Poelman 2022), we only used caterpillars that pupated successfully.

## 2 Materials and Methods

### 2.1 Caterpillar Sampling

**FIGURE 2:**
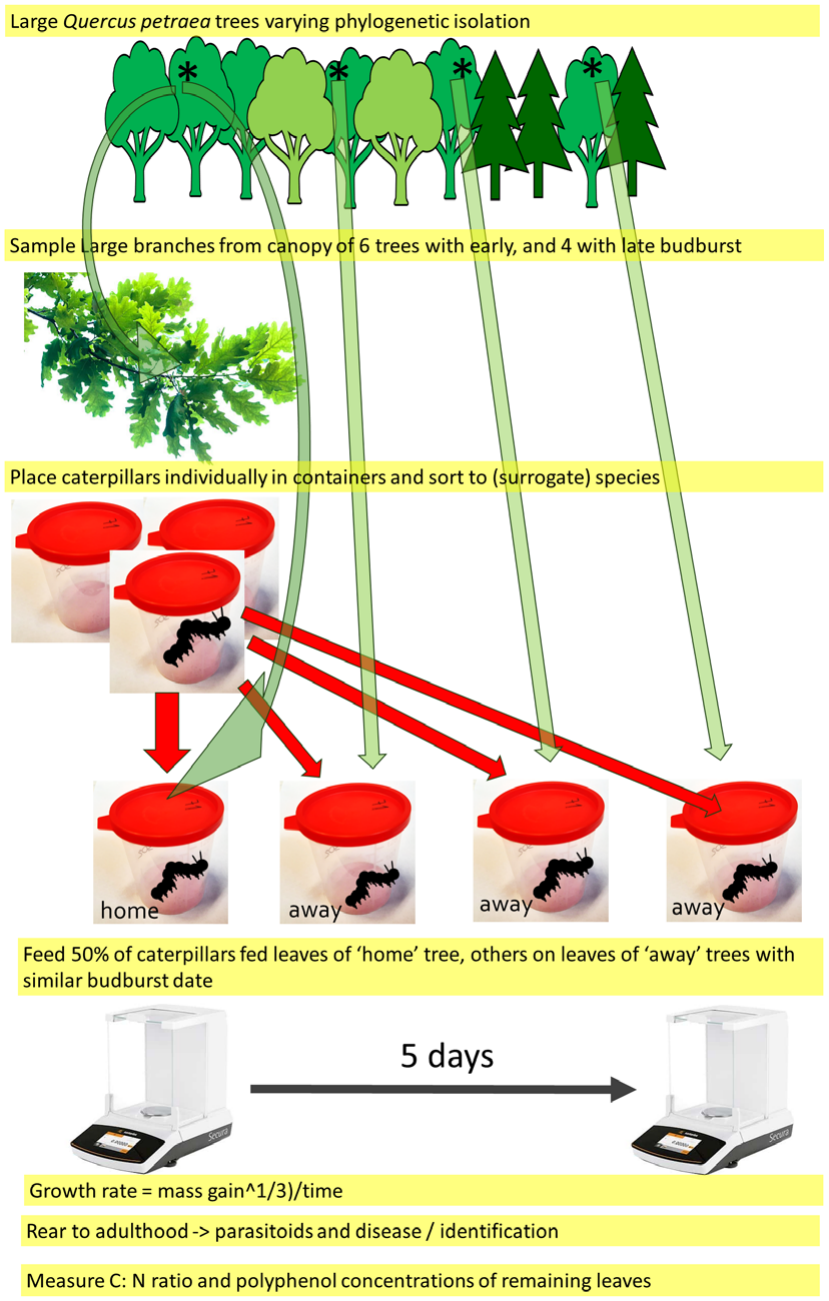
Experimental set‐up of the reciprocal transplant experiment showing only caterpillars originating from one of the focal trees and only for the late budburst set (four trees). The symbol * denotes sampled canopy branches from the canopy of focal trees.

To obtain caterpillars and leaves, we collected branches from the canopy of 10 sessile oak trees *Q. petraea* (Matt.) Liebl. in Puszcza Zielonka forest in Western Poland (Figure 2) using a slingshot and ropes. *Q. petraea* (and the closely related *Q. robur* L.; Jurkšienė et al. 2020) tend to show large inter‐individual differences in leaf traits (Kitamura et al. 2007; Roslin et al. 2006) and are known to harbor a wide variety of herbivorous insects (Southwood et al. 2005; Southwood 1961). The tree‐species composition in most compartments of this forest is dominated by pine ( *Pinus sylvestris* L.) or sessile oak ( *Q. petraea* ), while some smaller areas have mainly beech ( *Fagus sylvatica* L.), pedunculate oak ( *Q. robur* L.), or spruce ( *Picea abies* (L.) H. Karst.; Forest_Data_Bank 2018), and in some areas, there is a subcanopy of hornbeams ( *Carpinus betulus* L.; Forest_Data_Bank 2018). From the set of oak trees studied during previous years (Molleman and Walczak 2024; Molleman et al. 2022), we selected two sets of trees with similar budburst phenology (named ‘early budburst’ and ‘late budburst’), so that the stage of leaf development was similar within a set. Budburst phenology for the study year was estimated based on observations in 3‐day intervals (methods as in Molleman et al. 2022). Specifically, we sampled six trees on May 11^th^ 2022 (early budburst), and four trees on May 16^th^ 2022 (late budburst). In each set, the degree of phylogenetic isolation from the neighbors (average phylogenetic crown distance between the focal tree and trees with which its crown was in contact, see e.g. Mallick et al. 2023 for details and references) was selected to vary from low (most neighbors were oaks) to intermediate (mix of oak, pine, hornbeam, or beech) and high (surrounded mainly by pines, Table 1). The focal trees were widely dispersed in the study area (up to 8 km, Table 1). Notably, the nearest oak was never more than 50 m away, even if the distance between studied oaks was much larger. Thus, we compared the performance of caterpillars on the home tree to that on other trees in the forest, not to performance on neighboring trees. We cut the collected branches into pieces and transported them to the laboratory in plastic bags (4–6 60‐l bags per tree) and kept them overnight at a temperature of about 20°C.

**TABLE 1:** Location, diameter, and neighborhood of study trees.

Budburst	Tree	Site	Compartment	North	East	DBH	Oaks	Pines	Hornbeams	Beeches	PI
Early	27	Zielonka	123a	52.54068	17.12975	42	0	7	0	0	129.3
	28			52.54005	17.12907	43	0	8	0	0	140.0
	30			52.53933	17.12985	46	5	2	0	0	40.0
	34		115 h	52.54627	17.12312	50	2	6	0	0	105.0
	35			52.5461	17.12331	40	2	6	0	0	105.0
	45		123a	52.32441	17.08754	60	5	0	0	0	0.0
Late	13	Plawno	64 h	52.51511	17.09431	59	3	2	2	0	55.4
	15			52.51556	17.09366	66	2	3	1	0	79.0
	43	Pławno	52 m	52.31162	17.0556	32	0	4	0	2	106.7
	24	Kaminsko	73b	52.52544	17.05574	49	2	3	2	0	75.4

*Note:* Phylogenetic isolation was calculated from the number of oaks ( *Q. petraea* ) and other trees with which the focal *Q. petraea* was in contact.

Abbreviations: DBH = diameter at breast height, PI = phylogenetic isolation.

### 2.2 Caterpillar Rearing

The day after sampling, we searched the branches for caterpillars, and each caterpillar was placed individually in a 130 mL transparent cup with a red snap cap (Figure 2). We included both semi‐concealed (shelter builders such as leaf rollers) and free‐feeding caterpillars. We excluded *Carcina quercana* (Fab.) (Peleopodidae) caterpillars as our pilot study showed them to be very small and slow‐growing. We also excluded any caterpillars that appeared unhealthy or parasitized (e.g., spots, low activity). For each tree, we rapidly sorted the remaining caterpillars to species ( *Operophtera brumata* (L.), Geometridae; and *Favonius quercus* (L.), Lycaenidae) or surrogate species. We excluded singletons because we needed at least one ‘home’ and one ‘away’ individual for each species from a given tree. We assigned the remaining caterpillars to feed on trees of the set so that for each caterpillar (surrogate) species, about half would feed on the tree from which it was collected (home tree) and the remaining caterpillars were divided evenly over the other trees from the set (away trees). To obtain food for the caterpillars, on the day of caterpillar searching, we placed shoots from the trees into labeled plastic bottles with water and placed these in a growth chamber at 10°C with natural light:dark hours of 14 L:8D. We kept all cups with caterpillars on a wire tray inside one growth chamber with a constant temperature of 20°C and humidity of 75% with 14 L:8D. For the caterpillars from trees with early budburst, the caterpillars were left without food overnight and weighed the next morning on a semi‐micro balance (Sartorius, Secura 125‐1S, Goettingen, Germany) to an accuracy of 0.01 mg. Subsequently, we provided the caterpillars with a leaf from the tree they were assigned to and placed them back at 20°C. For the caterpillars from trees with late budburst, caterpillars were weighed the same evening after they were assigned to their diets and fed the next morning (which was possible due to the lower number of caterpillars). Thus, the second group spent slightly less time without food. Two days after the first feeding, we removed the frass and placed a new leaf from the assigned food tree inside each cup. Larger caterpillars were given more leaf material to ensure that they did not starve. Three days after that (5 days after the first feeding), we removed the remains of the leaves, and within 3 h, we weighed caterpillars as they were transferred to a new cup. Therefore, each caterpillar was weighed twice, providing data on mass gain over a five‐day period (Figure 2). The development time of small spring‐feeding Lepidoptera is often less than 2 weeks so that 5 days captures an important portion of their growth, while few individuals pupated within this time and would thus be lost to us (caterpillars typically lose a large and variable proportion of body mass during pupation; Molleman et al. 2011). To limit position effects, we randomized the position of cups on trays and trays inside the growth chamber at each handling. Subsequently, we reared caterpillars to pupation/parasitoid exit using locally obtained *Q. petraea* leaves. When all caterpillars had completed development or had died, we kept the pupae in the incubator at room temperature for 1 year. We identified all eclosed Lepidoptera using dissection of genitalia when needed (Lepiforum 2020; Razowski 1983, 1987; Wheeler 2023).

### 2.3 Leaf Phenolics and C:N Analysis

To measure aspects of leaf chemistry that are potentially important determinants of caterpillar growth on oak leaves (Feeny 1968; Forkner et al. 2004), we performed polyphenol analyses and measured the carbon–nitrogen ratio of leaves. To obtain samples for this analysis, after searching for caterpillars and setting shoots aside for feeding, we selected the remaining intact leaves (Figure 2) and dried them for 3 days at 35°C (POL‐EKO, SLW 400 STD, Wodzisław Śląski, Poland). We then cut the dried leaves in a blender and milled them using an IKA mill MF 10.1 (IKA‐Werke GmbH, Staufen, Germany). We dried a sub‐sample for C:N analysis for 24 h at 65°C. We used the Folin–Ciocalteu method (details in Molleman et al. 2024) to measure total phenolic content and measured antioxidant activity as Trolox equivalents (Gao et al. 2000; Re et al. 1999). The content of individual (classes of) phenolics was determined using an optimized high‐performance liquid chromatography coupled photodiode array (HPLC‐PDA) method (see Molleman et al. 2024; Appendix S1 for a detailed description of chemical methods). We quantified flavan‐3‐ols, flavonols, tannins, phenolic acids, and summed them up (we did not analyze them individually to avoid multiple testing or overparameterized multivariate models given the small sample size). Carbon and nitrogen contents (% dry mass) were determined using a 2400 CHN Elemental Analyzer (Perkin Elmer, Waltham, MA, USA).

### 2.4 Data Analysis

#### 2.4.1 Linking Caterpillar Morpho Species to Lepidoptera Species

On the one hand, caterpillars of a given species of Lepidoptera can have color polymorphism or change appearance across instars, while on the other hand, caterpillars of different species may look very similar. As a result, rapid sorting of caterpillars into surrogate species inevitably leads to errors. We matched the caterpillar surrogate species to positively identified adults and found that some caterpillar surrogate species belonged to the same species of Lepidoptera, while other caterpillar surrogate species could develop into multiple species of Lepidoptera (Appendix S1). Based on these links, we assigned caterpillars that did not reach adulthood to inferred species based on their surrogate species, where caterpillar surrogate species were assigned to an inferred species when at least 95% of them turned out to belong to a given species of Lepidoptera (Appendix S1).

#### 2.4.2 Local Adaptation

**FIGURE 3:**
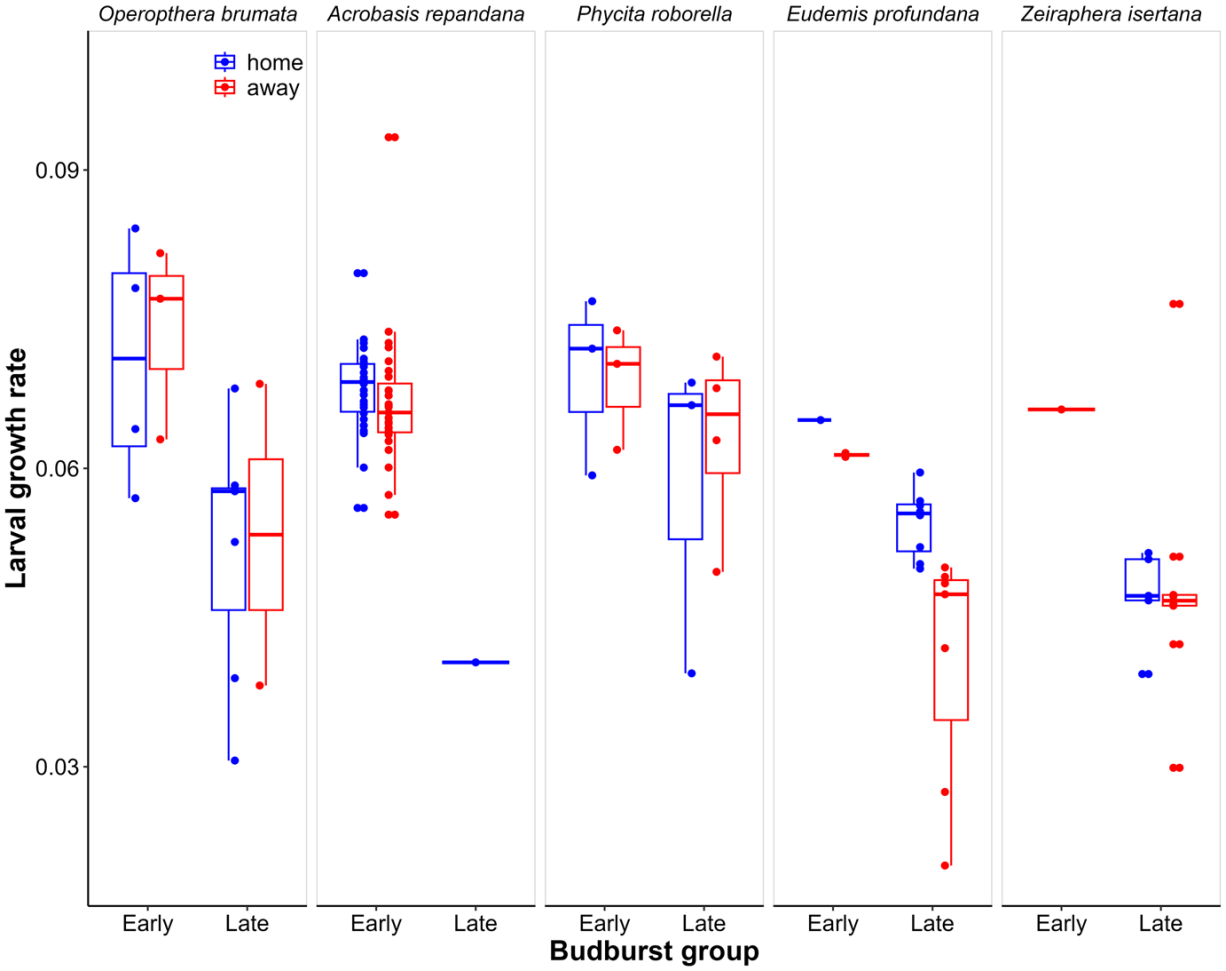
Scatter plots with boxplots of growth rate of caterpillars that pupated, for those fed on leaves from the tree they were collected from (home) vs. those that were fed on other trees of their set (away) per budburst group. The centre line denotes the median value (50th percentile), while the box contains the 25th to 75th percentiles and the black whiskers mark the 5th and 95th percentiles and outliers are denoted by dots (appearing as horizontal pairs). Details on the species are provided in Table 1 and statistical results are given in Table 3.

If caterpillars are adapted to the particular tree they originated from, we expected them to grow more rapidly when fed leaves from their home tree (home) than when fed on leaves from other trees (away). Therefore, we compared the growth rate of caterpillars that fed on the home vs. away trees. We excluded caterpillars that died (including those that were parasitized), so we analyzed those that pupated, irrespective of whether the adult eclosed from the pupa. We also excluded caterpillars that appeared to have lost body mass over the five‐day period (probably due to disease or weight loss associated with pupation). We calculated growth rate as ‘mass gain (0.3333)/time’ using grams and days, based on growth curves of caterpillars (Tammaru and Esperk 2007). We performed data visualization and analysis in R version 4.4.0 (R_Core_Team 2024). We first plotted growth rate on home vs. away trees for each species for the set of early and late bursting trees separately using ggplot2 (Wickham 2016). We then implemented linear models with as dependent variable larval growth rate, and as predictors home vs. away, early vs. late budburst, phylogenetic isolation, and the leaf traits C:N ratio, antioxidant activity (AOA), and concentrations of all polyphenols, flavonols, phenolic acids, flavan‐3‐ols, and tannins. Initial body mass was also included in the model, even though our formula for calculating growth rate should make this parameter relatively robust to variation in caterpillar size at collection. The identity of the food tree was first included as a random effect in a mixed model (lme4 package; Bates et al. 2011), but food tree explained none of the variation and was then included as a factor in general linear models (Appendix S3). We also included the interactions between home vs. away and phylogenetic isolation (neighborhood affecting local adaptation) and between home vs. away and species (local adaptation differing between species) as well as between species and leaf traits (species responding differently to leaf traits). We then performed model selection based on the Akaike information criterion corrected for sample size (AICc) using the dredge function of the package MuMIn (Barton and Barton 2015). Thus, the full model had as dependent variable larval growth rate, and as predictors home vs. away, inferred species, initial mass, early vs. late budburst, phylogenetic isolation of the tree of origin, the initial mass of the caterpillar, and the leaf traits C:N ratio, antioxidant activity (AOA), and concentrations of all polyphenols, flavonols, phenolic acids, flavan‐3‐ols, and tannins, initial body mass, and food tree as well as the interactions between initial mass and inferred species, home vs. away and inferred species, home vs. away and tannins, phylogenetic isolation (neighborhood affecting local adaptation) and between home vs. away, inferred species and tannins, inferred species and carbon–nitrogen ratio, and inferred species and phenolic acids. We evaluated the assumptions of the linear models, including normality of residuals, homoscedasticity, and independence of errors. Multicollinearity among predictors was assessed using Variance Inflation Factor (VIF), and no issues were detected in the final model (all VIF values < 7, Table A3.1 in Appendix S3). The different classes of polyphenols tended to be positively correlated with each other and negatively with C:N ratio (Figure A3.1 in Appendix S3). Figure 3 indicated that one species, *E. profundana* (Den. & Schiff.), showed local adaptation as this is the only species that shows on average higher growth rates on home trees. To explore this more, we implemented a separate model selection for this species, and also for the other species.

## 3 Results

### 3.1 Overview of the Data

We collected 567 caterpillars from the 10 trees and excluded 212 that were judged to be weak or parasitized, or were singleton (surrogate) species on a tree (Appendix S2). After matching surrogate species with identified adults (Appendix S1), removing individuals that lost body mass, excluding caterpillars that did not reach the pupal stage, and excluding rare species (*N* < 10), we were left with 115 individuals belonging to five species that could be used to test for local adaptation and the effects of leaf chemistry on caterpillar growth rate (Table 2, Figure 3).

**TABLE 2:** Characteristics of the five species of external‐feeding Lepidoptera for which we obtained a sufficient sample size to perform statistical analyses (*N* > 10).

Species	*N*	Family	Type	Winged ♀	Host plant	Wingspan (mm)
*Operophtera brumata*	16	Geometridae	Free‐feeding	No	Generalist	25.0
*Acrobasis repandana*	54	Pyralidae	Semi‐concealed	Yes	Oak	22.5
*Phycita roborella*	13	Pyralidae	Semi‐concealed	Yes	Mainly oak	26.5
*Eudemis profundana*	17	Tortricidae	Semi‐concealed	Yes	Oak	18.0
*Zeiraphera isertana*	15	Tortricidae	Semi‐concealed	Yes	Mainly oak	15.5

*Note:* Wingspan of *O. brumata* is for males.

### 3.2 Adaptation to Individual Trees

Overall, caterpillars fed on leaves of home trees were growing faster than those fed on leaves from away trees (Table 3). This appeared fully attributable to one species, *E. profundana*, that grew faster on home trees than on away trees (Figure 3, Table 3). This indicates that only this species may have been adapted to the leaf traits of the home trees. Phylogenetic isolation did not appear to affect this local adaptation as it was not retained in the AICc‐selected model (Table 3). Overall, caterpillars with a larger initial mass tended to grow faster (Table 3). When initial mass was taken into account, species identity was not selected into the models, as larger‐bodied species tended to have higher growth rates. Caterpillars from the early budburst set tended to grow faster than those from the late budburst set (Figure 3, Table 3). Most of the leaf traits (other than flavonols) were not selected into the model, and the effect of flavonols was also not significant (Table 3). The second‐best model (delta AICc = 0.07) included tannins instead of flavonols, and the third model (delta AICc = 0.71) had none of the leaf chemistry parameters (Table A3.2 in Appendix S3). None of the interaction terms were included in the AICc‐selected model (Appendix S3).

**TABLE 3:** Results of the general linear model selected using AICc model and a separate selected model for the species showing signs of local adaptation, *Eudemis profundana* (Figure 3).

Predictors	Estimate	SE	*T*	*p*
Selected model				
Home versus away	−0.004	0.002	−2.036	**0.044**
Starting mass	0.283	0.093	3.052	**0.003**
Budburst set	0.028	0.005	5.917	**< 0.001**
Flavonols	0.000	0.000	−1.687	0.094
Selected model for *Eudemis profundana*				
Home versus away	−0.014	0.004	−3.263	**0.006**
Budburst set	0.021	0.007	3.149	**0.007**

*Note:* The dredge results of the top 50 models are given in Appendix S3 Table A3.2.

*P*‐values are in bold when they are smaller than 0.05.

## 4 Discussion

We performed a reciprocal transplant experiment on a caterpillar community on *Q. petraea* and found that the overall faster growth rate on home trees was attributable to one species, so that only one out of five species showed evidence for adaptation to leaf traits of individual trees. This local adaptation was not affected by phylogenetic isolation. However, the species for which we do find a significant increase of growth rate on home trees is not the most abundant. There are three other Lepidoptera species that are similarly or more abundant, and even qualitatively the patterns in these species do not go in the direction of increased growth rate on home trees. Therefore, it appears that pronounced local adaptation is the exception rather than the rule among caterpillars in our model system and study year.

### 4.1 Why Is Local Adaptation Rare in Caterpillars?

The overall lack of a home vs. away effect may be explained by insect movements between trees (no local populations), weak selection by tree traits other than budburst phenology, and population history (not enough time to adapt since colonization). External‐feeding Lepidoptera may not often form populations at the level of individual trees, but instead disperse across trees in the forest yearly. That specifically *E. profundana* showed local adaptation may be because it was among the smallest‐bodied species in our study, and may thus be one of the least apt to disperse. Limited dispersal is also expected for *O. brumata*, which has wingless females. However, this is a host‐plant generalist, which appeared to have recolonized *Q. petraea* from hornbeams at most three years prior to the experiment (Molleman and Walczak 2024), probably due to frost after oak budburst in the spring of 2018. Therefore, local adaptation to tree‐specific leaf characteristics may not be likely in this species during the study year. Other species may have suffered a similar calamity and may thus have recolonized the trees too recently to show local adaptation (Mopper et al. 2000). More generally, the importance of local adaptation may vary over time. Additionally, trees may need to be more isolated than those in our study (where other oak trees always occurred in the stand, even when none of the neighbors were oaks) to allow these insects to become locally adapted.

Even if moths form local populations, they may not show significant local adaptation. Since leaf quality changes dramatically over time during spring, variation in budburst phenology and speed of leaf development may be more important than particular polyphenol profiles (Gripenberg et al. 2007; Lindroth et al. 2024; Salminen et al. 2004). Indeed, *O. brumata* can show adaptation to the budburst phenology of individual trees (Van Dongen et al. 1997), and this may also be the case in other species of moths (Du Merle 1988). In our experiment, we tried to exclude the effect of budburst phenology by using sets of trees that are similar in this respect to focus on adaptation to leaf characteristics such as C:N and polyphenols. Local adaptation may also be hindered by variation among years in leaf palatability of trees such as those found in *Q. robur* (Ruhnke et al. 2009).

## 5 Conclusions

We found limited evidence for local adaptation in response to traits of leaves (our design attempted to exclude effects of budburst phenology) in lepidopteran caterpillars, with one out of five species (*E. profundana*) showing significantly higher growth rates on home trees compared to away trees. Possible explanations for this are that such insects do not often form populations at the level of individual trees, that any local adaptations may be swamped by new arrivals, or that leaf traits of individual trees exert little selection on these herbivores. However, this may vary among years, forests, caterpillar species, and host tree species. Furthermore, adaptation to for example, budburst phenology of individual trees may be more common than local adaptation to leaf chemistry. Since phylogenetic isolation did not affect the degree of local adaptation in our study, local adaptation to leaf traits does not appear to be important in explaining the effects of phylogenetic isolation on herbivory by Lepidoptera.

## Author Contributions

**Freerk Molleman:** conceptualization (equal), data curation (lead), formal analysis (lead), funding acquisition (lead), investigation (equal), methodology (equal), supervision (lead), writing – original draft (lead), writing – review and editing (equal). **Anna Sokół‐Łętowska:** data curation (supporting), investigation (equal), writing – review and editing (supporting). **Soumen Mallick:** investigation (equal), writing – review and editing (supporting). **Andreas Prinzing:** conceptualization (supporting), formal analysis (supporting), writing – review and editing (supporting). **Urszula Walczak:** conceptualization (equal), data curation (supporting), funding acquisition (supporting), investigation (equal), methodology (equal), project administration (lead), writing – review and editing (equal).

## Conflicts of Interest

The authors declare no conflicts of interest.

## Supporting information


**Appendix S1.** Matching caterpillar surrogate species to identified moths.

**Appendix S2.** Growth rate dataset.

**Appendix S3.** Statistical details.

## Data Availability

Data have been made available as an appendix S1. We are open to posting our data on Dryad.
