# Supplementary material for: Adaptation to Leaf Traits of Individual Trees in a Forest Appears Rare in Caterpillars
Source: Ecol Evol. 2025 Mar 13;15(3):e71038. doi: 10.1002/ece3.71038 (PMC11904093; doi:10.1002/ece3.71038)
Supplement: Supplementary file 3 — Appendix S3. Statistical details. [file ECE3-15-e71038-s003.docx]

**Appendix 3. Statistical Details**

Table A3.1. Variance Inflation Factors for the selected model.

HomeAway Weight1 Batch flavonols

1.068 1.174 6.001 5.841

**
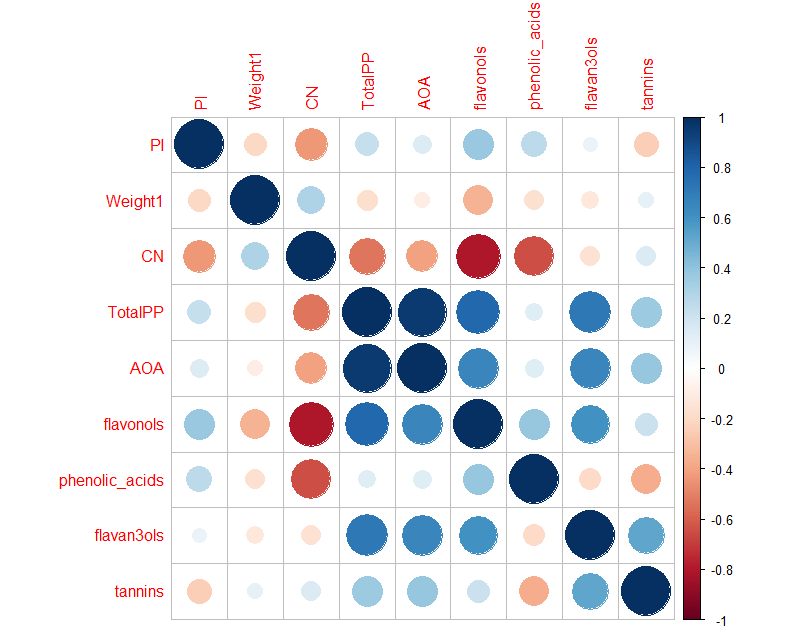
**

Figure A3.1. Correlogram of predictors of caterpillar growth rate used in the full model. PI = Phylogenetic Isolation, Weight1 = initial mass of caterpillar, CN = carbon-nitrogen ratio, TotalPP = total polyphenol concentration, AOA = anti-oxidant activity.


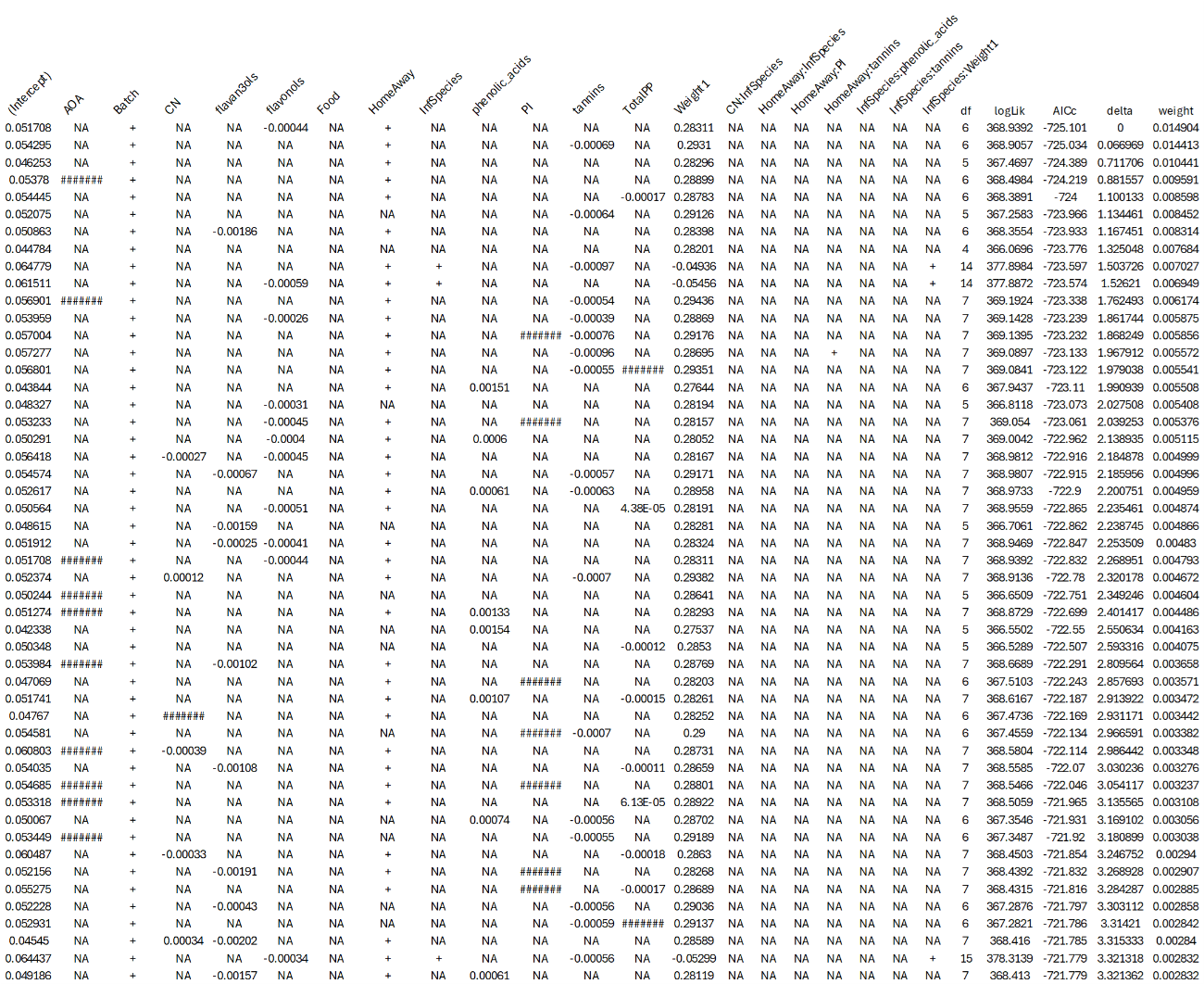


Table A3.2. The top 50 models based on AICc found using Dredge for larval growth rate.

Groups Variance Std.Dev.

Food 0.000000 0.0000

Residual 0.000104 0.0102

Table A3.3. Results for the random effect when food tree is included a s random effect in the selected model.

Number of obs: 117, groups (Food): 11
